# Data on chromatographic isolation of cysteine mixed-disulfide conjugates of Allium thiosulfinates and their role in cellular thiol redox modulation

**DOI:** 10.1016/j.dib.2018.10.144

**Published:** 2018-11-01

**Authors:** Restituto Tocmo, Kirk Parkin

**Affiliations:** Department of Food Science, University of Wisconsin-Madison, Babcock Hall, 1605 Linden Drive, Madison, WI 53706, United States

## Abstract

This data article contains experimental data on the preparation and semi-preparative isolation of *S*-Alk(en)ylmercaptocysteine (CySSRs, R = allyl, “A”, 1-propenyl, “Pe” or methyl, “Me”) generated through conjugation reactions between allyl and 1-propenyl enriched thiosulfinates (TS) and cysteine. The data presented are related to the research article “*S-*Alk(en)ylmercaptocysteine suppresses LPS-induced pro-inflammatory responses in murine macrophages through inhibition of NF-κB pathway and modulation of thiol redox status” (Tocmo and Parkin, in press). In this data article, we included a detailed procedure for CySSR preparation, their purification through semi-preparative chromatography and their toxicity profiles in RAW 264.7 macrophages. Data included also highlight, the ability of CySSRs to modulate intracellular thiol redox status.

**Specifications table**

**Table:** 

Subject area	*Redox biology*
More specific subject area	*Organosulfur chemistry and cellular thiol redox status*
Type of data	*Figures and flow charts*
How data were acquired	*Reveleris® Prep 134 purification system, microplate reader* (Molecular Devices)
Data format	*Raw and analyzed*
Experimental factors	*Cells were treated with lipopolysaccharides (LPS) or L-Buthionine-sulfoximine (BSO) with or without CySSRs*
Experimental features	*CySSRs were prepared and isolated using a semi-preparative chromatographic system. Dose- and time-dependent responses upon treatment either with CySSRs, LPSor BSO were measured.*
Data source location	*Department of Food Science, University of Wisconsin-Madison, Madison, WI, USA 53706.*
Data accessibility	*All data are available in this document.*
Related research article	*R. Tocmo, K. Parkin, S-Alk(en)ylmercaptocysteine suppresses LPS-induced pro-inflammatory responses in murine macrophages through inhibition of NF-κB pathway and modulation of thiol redox status, Free Radic. Biol. Med., 2018. In press*[1]

**Value of the data**

• The method and data can be used to obtain 1-propenyl-enriched TS extracts from Alliums.
• The data can be used to isolate highly pure 1-propenyl bearing CySSR species.
• The data provide information on intracellular thiol redox-modulating action of CySSRs and on their cytotoxicity in RAW 264.7 cells that may be used as reference information for further bioactivity explorations.

## 1 Data

**Fig. 1:**
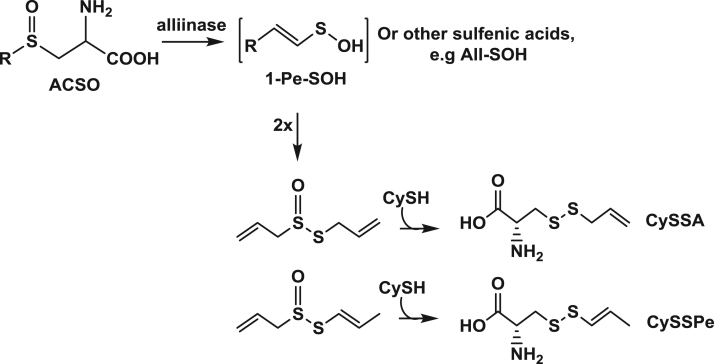
Mechanism of *S*-Alk(en)ylmercaptocysteine formation.

**Fig. 2:**
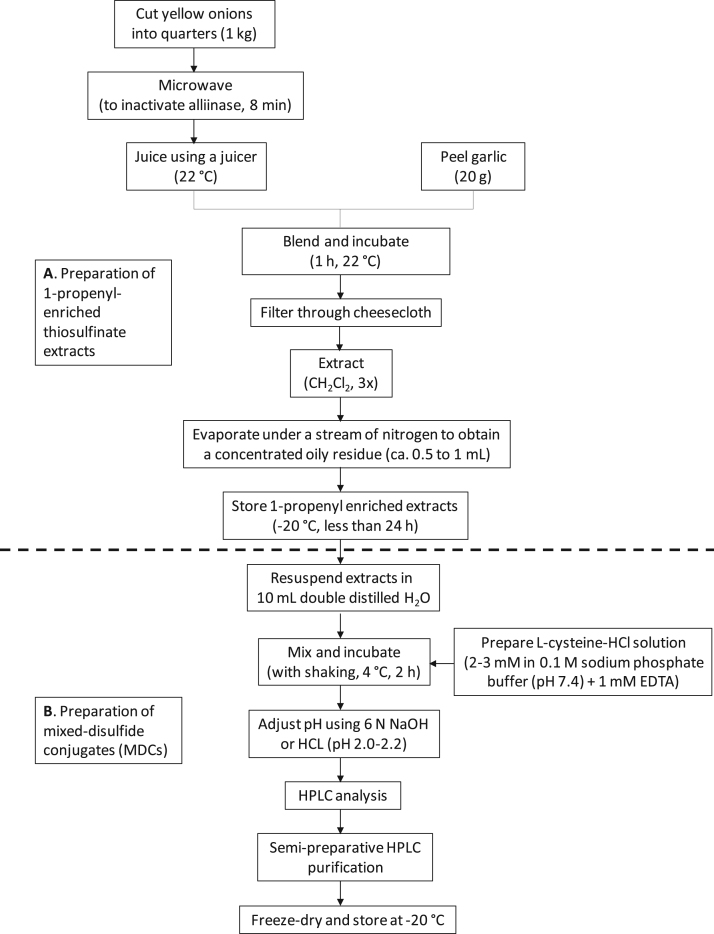
Extraction of 1-propenyl-enriched thiosulfinate extracts and preparation of *S*-Alk(en)ylmercaptocysteines.

**Fig. 3:**
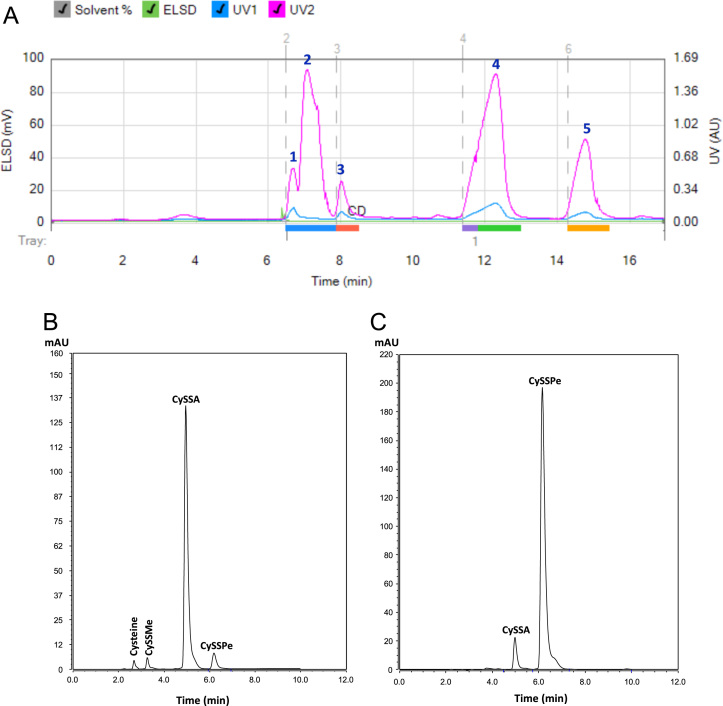
Representative chromatograms of (A) crude CySSR and purified (B) CySSA and (C) CySSPe. The analytical conditions are described in the materials and methods part [1], [2], [3]. Peaks: 1, cysteine; 2, cystine; 3, CySSMe; 4, CySSA; 5, CySSPe.

**Fig. 4:**
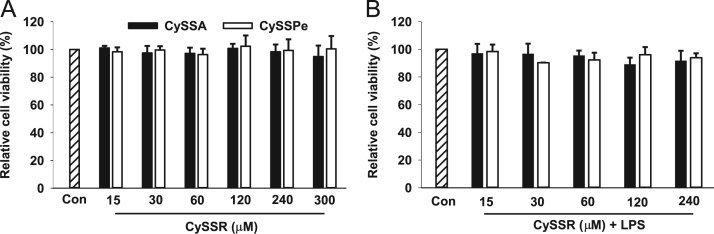
CySSR did not affect viability of RAW 264.7 cells. Cells were incubated with (A) CySSRs (15–240 μM) only or with (B) CySSRs plus LPS (1 μg/ml) or for 24 h, and the cell viability was determined by the MTT assay [1].

**Fig. 5:**
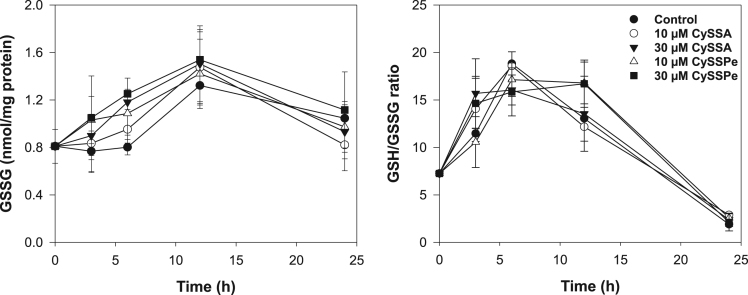
Time-dependent changes in GSSG levels. Cells were incubated with 10 and 30 µM of CySSRs for 3, 6, 12, and 24 h, after which GSH [1,Fig. 7] and (A) GSSG levels were measured. GSH and GSSG values were used to calculate the (B) GSH:GSSG ratio. Assay conditions were described in the materials and methods part [1].

**Fig. 6:**
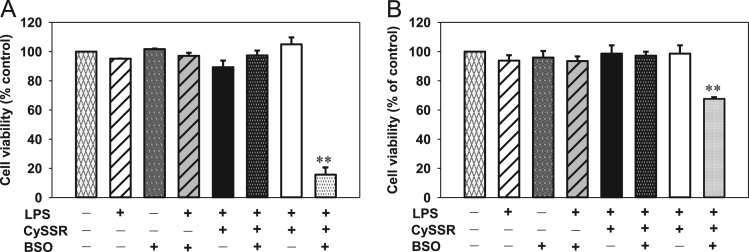
Cell viability after CySSR exposure of BSO pre-treated LPS-induced macrophages. Cells were incubated with BSO (50 μM) for 18 h followed by co-incubation with LPS (1 μg/ml) + CySSRs (60 μM) + BSO (50 μM). Cell viability after 24 (A, for NO measurement) or 6 h (B, for ROS measurement) was determined by the MTT assay [1].

The data presented include an overview of the mechanism of CySSR formation (Fig. 1), a flow chart for CySSR preparation (Fig. 2) and the chromatographic profiles of CySSRs separated and isolated through high performance liquid chromatography (HPLC) and semi-preparative chromatography, respectively (Fig. 3a–c). The cytotoxicity of CySSR in non-activated and LPS-induced RAW 264.7 macrophages is presented in Fig. 4a and b. The effects of CySSRs on the intracellular levels of oxidized glutathione (GSSG) and its ratio relative to reduced glutathione levels (GSH) are shown in Fig. 5a and b. The data on cell viability after BSO pre-treatment and subsequent CySSR exposure are presented in Fig. 6a and b.

## 2 Experimental design, materials and methods

The data provided illustrate a step-by-step procedure for obtaining 1-propenyl-enriched TS extracts. 1-propenyl-enriched TS extracts were obtained following a previously reported method [2,3], with some modifications (Fig. 1). After conjugation reactions between TS extracts and cysteine-HCl, the CySSRs formed were isolated [1] to obtain highly pure (>90%) CySSA and CySSPe and the chemical structures and identities of the isolated compounds were confirmed by comparing their LC-MS and NMR spectra to those previously reported [2], [3]. The cytotoxicity profiles of isolated pure conjugates in RAW 264.7 cells were determined using a standard MTT assay. CySSRs were also evaluated for their role in modulating thiol redox status in RAW macrophages by measuring intracellular levels of reduced (GSH) and oxidized (GSSG) glutathione, and the GSH:GSSG ratio [1].
